# Collagen peptide supplementation in combination with resistance training improves body composition and increases muscle strength in elderly sarcopenic men: a randomised controlled trial

**DOI:** 10.1017/S0007114515002810

**Published:** 2015-10-28

**Authors:** Denise Zdzieblik, Steffen Oesser, Manfred W. Baumstark, Albert Gollhofer, Daniel König

**Affiliations:** 1 Department for Nutrition, Institute for Sports and Sports Science, University of Freiburg, Freiburg 79117, Germany; 2 CRI, Collagen Research Institute GmbH, Kiel 24118, Germany; 3 Department of Rehabilitation, Prevention and Sports Medicine, Centre for Internal Medicine, University Hospital Freiburg, 79106 Freiburg, Germany

**Keywords:** Sarcopenia, Collagen hydrolysate, Collagen peptides, Resistance exercise, Ageing, Protein supplementation

## Abstract

Protein supplementation in combination with resistance training may increase muscle mass and muscle strength in elderly subjects. The objective of this study was to assess the influence of post-exercise protein supplementation with collagen peptides *v.* placebo on muscle mass and muscle function following resistance training in elderly subjects with sarcopenia. A total of fifty-three male subjects (72·2 (sd 4·68) years) with sarcopenia (class I or II) completed this randomised double-blind placebo-controlled study. All the participants underwent a 12-week guided resistance training programme (three sessions per week) and were supplemented with either collagen peptides (treatment group (TG)) (15 g/d) or silica as placebo (placebo group (PG)). Fat-free mass (FFM), fat mass (FM) and bone mass (BM) were measured before and after the intervention using dual-energy X-ray absorptiometry. Isokinetic quadriceps strength (IQS) of the right leg was determined and sensory motor control (SMC) was investigated by a standardised one-leg stabilisation test. Following the training programme, all the subjects showed significantly higher (*P*<0·01) levels for FFM, BM, IQS and SMC with significantly lower (*P*<0·01) levels for FM. The effect was significantly more pronounced in subjects receiving collagen peptides: FFM (TG +4·2 (sd 2·31) kg/PG +2·9 (sd 1·84) kg; *P*<0·05); IQS (TG +16·5 (sd 12·9) Nm/PG +7·3 (sd 13·2) Nm; *P*<0·05); and FM (TG –5·4 (sd 3·17) kg/PG –3·5 (sd 2·16) kg; *P*<0·05). Our data demonstrate that compared with placebo, collagen peptide supplementation in combination with resistance training further improved body composition by increasing FFM, muscle strength and the loss in FM.

In general, ageing is associated with a decline in motor function, muscle mass and a decrease in muscular performance^(^ ^1^ ^,^ ^2^ ^)^. The definition of sarcopenia includes both an age-related decline in muscle mass and a reduction in functional muscular performance. Sarcopenia is associated with an increased risk for falls and an overall prevalence for frailty^(^ ^3^ ^,^ ^4^ ^)^. Several investigations have shown that the onset of sarcopenia can be postponed and the progress decelerated by regular physical activity, mainly resistance exercise^(^ ^5^ ^–^ ^7^ ^)^. Furthermore, it has been demonstrated that additional dietary proteins enhance the rate of post-exercise net muscle protein synthesis and decrease muscle protein breakdown following resistance exercise^(^ ^8^ ^–^ ^10^ ^)^. Consequently, the combination of prolonged resistance exercise and post-exercise protein supplementation should increase fat-free mass (FFM) and/or muscle strength in randomised controlled trials (RCT). However, although several well-controlled studies have shown an increase in strength or FFM, a comparable number of investigations have yielded negative results^(^ ^8^ ^,^ ^9^ ^)^. In a most recent meta-analysis, Cermak *et al.* ^(^ ^11^ ^)^ included twenty-two RCT that have investigated the effect of resistance exercise and protein supplementation on FFM and muscle strength in both young and older subjects. Their analyses showed that protein supplementation increases FFM and strength to a significantly higher level than placebo and that this effect of dietary protein was evident in both younger and older subjects. In most of these RCT, the proteins administered were whey, milk, soya or casein; in some studies, a mixture of different essential amino acids was administered.

In the present study, we investigated the effect of post-exercise protein supplementation with collagen peptides on muscle mass and muscle function during a 3-month resistance training programme. Collagen is an extracellular protein that accounts for 25–30 % of the total protein content within the human body. The process of hydrolysis yields collagen peptides that are designated as foodstuff. The peptides are rapidly resorbed in the small intestine, which may be important for post-exercise recovery, although the existence of the post-exercise metabolic window has recently been challenged^(^ ^12^ ^)^. Moreover, collagen peptides are absorbed in intact form to some extent, up to 10 kDa^(^ ^12^ ^–^ ^15^ ^)^.

It is generally believed that the protein applied should be high in branched chain amino acids (BCAA), particularly leucine, which is known to activate several intracellular signal transduction pathways involved in initiating translation such as the mTOR signalling pathway^(^ ^16^ ^,^ ^17^ ^)^.

Collagen is generally regarded as having a relatively low biological value, mainly due to the low amount of BCAA and lysine (Table 1). Nevertheless, the mixture of amino acids has been shown to be superior compared with whey protein in maintaining N balance and body weight during a low-protein diet^(^ ^18^ ^)^. In addition, collagen contains relatively high amounts of arginine and glycine, both known to be important substrates for the synthesis of creatine in the human body^(^ ^19^ ^)^.

**Table 1:** Amino acid composition of the collagen peptides

Amino acid	Weight (%)	Mol (%)
Hydroxyproline	11·3	9·6
Aspartic acid	5·8	4·8
Serine	3·2	3·4
Glutamic acid	10·1	7·5
Glycine	22·1	32·3
Histidine	1·2	0·8
Arginine	7·8	5·0
Threonine	1·8	1·7
Alanine	8·5	10·5
Proline	12·3	11·8
Tyrosine	0·9	0·5
Hydroxylysine	1·7	1·2
Valine	2·4	2·3
Methionine	0·9	0·9
Lysine	3·8	2·9
Isoleucine	1·3	1·1
Leucine	2·7	2·3
Phenylalanine	2·1	1·4

Although hydrolysed collagen is contained in sports drinks and bars aimed at improving regeneration and post-exercise muscle recovery, to our knowledge, no controlled study has thus far investigated the effect of collagen peptide supplementation on FFM, muscle strength and motor control. We investigated the respective effects in combination with resistance training in a randomised placebo-controlled design in fifty-three elderly men with sarcopenia class I and II.

## Methods

### Subjects

**Fig. 1:**
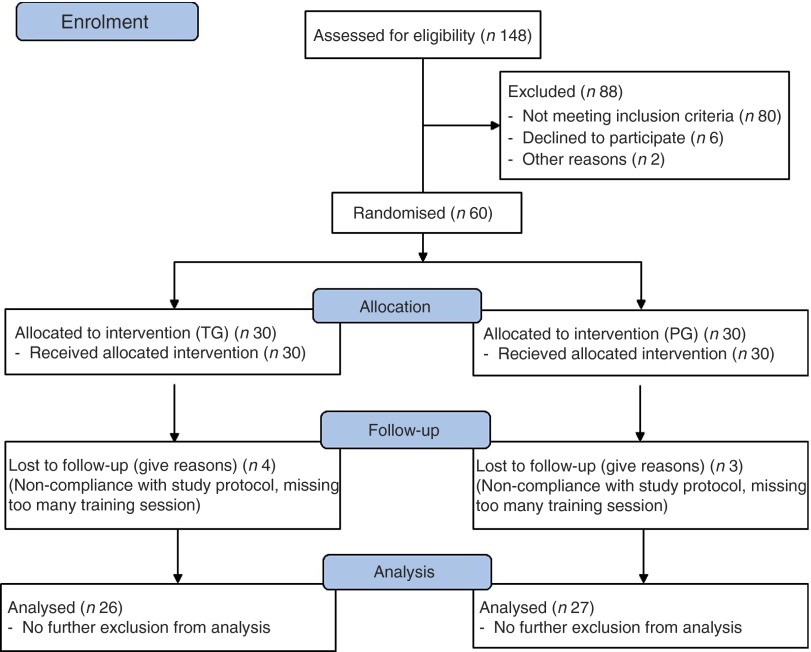
Flowchart of subject recruitment and dropouts before and during the study. TG, treatment group; PG, placebo group.

A total of 148 subjects (Fig. 1) answered an advertisement in a local newspaper in which healthy men, aged>65 years, who experienced a considerable loss in muscular strength or physical performance within the last 3–4 years, were sought. Subjects needed to be able to participate in the 3-month resistance training and be free of acute diseases or illness-related cachexia.

After a telephone interview, forty-two subjects already met the exclusion criteria. These subjects had chronic illnesses (liver, kidney, cancer without recurrence for 5 years, CVD, advanced arthrosis) or other diseases that made participation in the exercise programme impossible; 106 persons were then invited to attend our outpatient ward for further examinations. From these 106 subjects, again forty-six of them met the exclusion criteria, mainly because sarcopenia was absent or because the medical examination yielded further contraindications to participate in the resistance training programme. The presence of sarcopenia was screened using a handheld dynamometer (Trailite; LiteExpress GmbH). According to the European working group on sarcopenia in older people, handgrip strength (<32 kg) is well suited for detecting low muscle strength^(^ ^20^ ^)^. The presence of sarcopenia was then assured by dual-energy X-ray absorptiometry (DXA) measurement of muscle mass. The diagnosis and classification of sarcopenia were established by the loss of muscle mass and muscle function according to current guidelines^(^ ^20^ ^–^ ^22^ ^)^. Sarcopenia class I was diagnosed if DXA muscle mass was 1–2 sd lower than the sex-specific mean for young adults, and sarcopenia class II was diagnosed if the muscle mass was lower than 2 sd.

Among all, sixty subjects gave their written informed consent after being informed about the nature and the possible risks of the investigation. All the subjects completed a comprehensive medical examination and routine blood testing (ESR, haemogram, creatinine, creatine kinase, urea, ALT, AST) to exclude other chronic diseases.

The study protocol was approved by the ethics committee of the University of Freiburg.

### Study design

The participants of the study were randomly assigned to the treatment group (TG) (collagen peptide supplementation) or to the placebo group (PG). Randomisation was performed using a random number generator^(^ ^21^ ^)^. Blinding of investigators and participants was not lifted until all the data were entered, the data set was secured and the statistical analysis was performed.

The primary outcome measure was the change in FFM before and after the intervention, which lasted for 12 weeks.

Compliance was checked by collecting unused supplements. In addition, subjects were asked to keep daily records about the timing of ingestion, side-effects or other problems related to the training programme or the supplements. In addition, blood samples were collected at the beginning and at the end of the study to evaluate the safety of the product and to verify adverse reactions.

### Protein supplementation

The subjects assigned to the TG (*n* 30) were given 15 g of collagen peptides/d. The test product with a mean molecular weight of approximately 3 kDa is derived from a complex multi-step procedure by the degradation of type I collagen. The product was provided by GELITA AG (BODYBALANCE^™^). The amino acid composition of the collagen peptides is shown in Table 1.

Subjects in the PG (*n* 30) received silicon dioxide (Sipernat 350; Evonik). Silicon dioxide (silica) was chosen because it is a safe food additive and is absorbed in negligible amounts by the intestine. Therefore, silicon dioxide induces no metabolic effects in contrast to, for example, carbohydrates applied in some of the previous studies in this field.

Collagen peptides as well as placebo were given in powder form and were dissolved by the participants in 250 ml water. Subjects were instructed to drink the solution as soon as possible following each training session but not later than 1 h after training. During the first hour after training, no other food was allowed, except for water to compensate for sweat loss. Subjects also ingested collagen hydrolysate/placebo on the days without training; they were requested that the time point when they drank the solution without previous training should not differ from the days with training.

### Exercise intervention programme

The resistance training was carried out at the University of Freiburg and consisted of a 12-week guided training programme on fitness devices (pull down, leg press, bench press, back press, etc.) involving all larger muscle groups. Subjects took part in the resistance training programme in the afternoon three times a week over a time period of 60 min. Individual adaptations of the training protocol were regularly made as a function of the actual performance. The intensity was based on the number of possible repetitions (week 1–4: fifteen repetitions, week 5–9: ten repetitions, week 10–12: eight repetitions; 4 s/repetition). Subjects were excluded from the study if they missed >10 % of the training sessions.

### Methods

Body composition was measured before and after the 3-month training period using DXA (Stratos DR 2D Fan Beam; Degen Medizintechnik).

Muscular strength was tested by measuring isokinetic quadriceps strength of the right leg before and after the training programme (Con-Trex) and sensory motor control (SMC) was determined using a standardised one-leg stabilisation test (Posturomed; Haider-Bioswing) as described previously^(^ ^22^ ^)^.

### Dietary intake

Dietary intake was evaluated before and at the end of the study using 4 days’ nutritional protocols. Subjects were asked to fill out the protocols using household measurements. The protocols were analysed using PRODI 6.0 (Prodi).

### Statistical methods

All the data in the tables are presented as means and standard deviations and as means with their standard errors in the bar charts. Statistical analysis was performed using the Statistical Package for the Social Sciences Software (SPSS for Windows, version 20.0.1). Normality of all the variables was tested before statistical evaluation using the Kolmogorov–Smirnov test. All the variables were normally distributed. Baseline differences were tested using the unpaired samples *t* test. Testing for changes between examination at baseline and following the 3-month intervention within groups were performed using the paired samples *t* test. Testing for changes between groups following the intervention (collagen peptide group=TG *v.* PG) was carried out using two-way repeated-measures ANOVA for continuous variables. The factors were TG (collagen hydrolysate/placebo) and time (levels were pre- and post-intervention).

The strength of relationships was analysed using Pearson’s linear correlation coefficient *r*. A *P* value of 0·05 or less was considered to indicate statistical significance. Based on previous studies, we expected an increase in FFM (primary outcome measure) by 2 kg with a 2·5 sd ^(^ ^23^ ^)^. With an *α* of 0·05 and a power of 0·80, a number of twenty-five subjects in each group was considered appropriate. Considering a dropout rate from 20 %, we chose a number of thirty subjects in each group.

## Results

### Subjects

A total of fifty-three men with a mean age of 72·2 (sd 4·68) years completed the study (twenty-six men in the TG and twenty-seven men in the PG). Age did not differ significantly between the completers in both groups (TG=72·3 (sd 3·7) years and PG=72·1 (sd 5·53) years).

All seven dropouts were related to incompliance with the study design and training protocol. Excluded participants predominantly had missed >10 % of the training sessions due to various reasons. No dropout was related to side-effects of the administered collagen peptide supplement or placebo. No serious adverse events were noted and, especially, no pathological findings could be observed in the routine blood tests.

Based on the results of the handgrip test and the DXA measurements^(^ ^24^ ^)^, twenty-one subjects of the total study population were categorised as having class I sarcopenia and thirty-two as having class II sarcopenia. Again, data were balanced at baseline with no statistically significant differences between the TG and the PG, regarding classification of sarcopenia (TG=eleven class I and fifteen class II; PG=ten class I and seventeen class II).

### Body composition and muscle strength

In both the groups, a statistically significant (*P*<0·001) increase in FFM and a significant loss in fat mass (FM) (*P*<0·001) could be observed after 3 months (Table 2). Moreover, muscle strength and SMC improved significantly (*P*<0·001) in both the groups. Moreover, data for bone mass (BM) revealed a statistically significant (*P*<0·001) increase in both the groups at the end of the study.

**Table 2:** Body composition, muscle strength and sensory motor control in the subjects before and after supplementation with collagen hydrolysate or placebo (Mean values and standard deviations)

	Treatment group (*n* 26)				Placebo group (*n* 27)				
	Baseline examination		Final examination		Baseline examination		Final examination		
	Mean	sd	Mean	sd	Mean	sd	Mean	sd	Significance between groups in RM ANOVA testing assessing (treatment×time) interaction (*P*)
Weight (kg)	88·2†	12·1	87·3	11·9	83·1	14·8	82·8	14·5	NS
Fat-free mass (%)	64·7†	4·26	70·31***	4·8	66·8	4·77	70·4***	4·94	<0·05
Fat mass (%)	31·63†	4·58	25·67***	5·22	29·5	5·53	25·4***	5·55	<0·05
Bone mass (%)	3·6†	0·47	4·02***	0·59	3·81	0·61	4·18***	0·71	NS
Fat-free mass (kg)	56·9†	6·68	61·1***	6·88	54·9	6·96	57·8***	7·46	<0·05
Fat mass (kg)	28·1†	7·09	22·7***	7·08	25·1	8·69	21·6***	8·15	<0·05
Bone mass (kg)	3·14†	0·36	3·46***	0·38	3·1	0·36	3·34***	0·43	NS
Power (knee extension) (Nm)	123†	27·3	140***	28·3	132	27	139*	27·4	<0·05
Sensory motor control (mm)	1205†	852	477***	228	1374	639	516***	24	NS

RM, repeated measurements; mm, length of path on posturometer.

* *P*<0·05 within the group from baseline to final examination; ***P<0·001 within the group from baseline to final examination.† No significant difference at baseline between treatment group and placebo group.

**Fig. 2:**
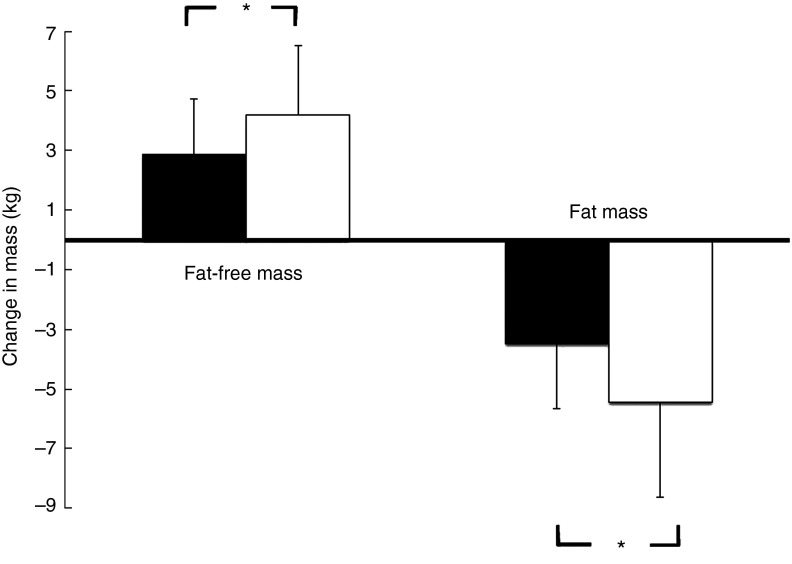
Change in fat-free mass and fat mass after 12 weeks of resistance training in elderly men (age>65 years) with collagen peptide supplementation (treatment group, *n* 26; 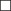) or placebo (placebo group, *n* 27; 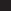). Values are means, with their standard errors represented by vertical bars. Significance was tested by ANOVA considering time × treatment interactions. * Mean value was significantly different from that of the placebo group (P<0.05).

**Fig. 3:**
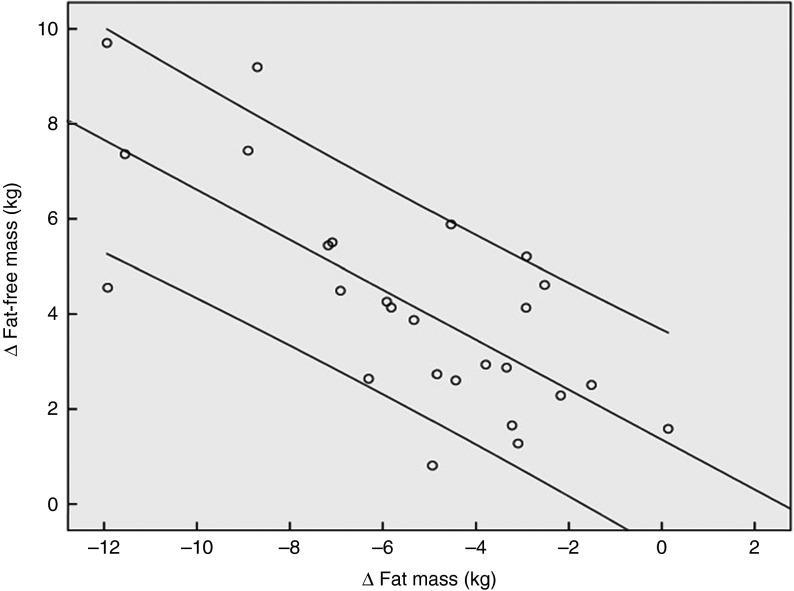
Correlation (Pearson’s *r*) between fat-free mass and fat mass changes after a 12 weeks of resistance training in elderly men (age>65 years, *n* 26) in combination with a daily dosage of 15 g collagen peptides (*r* 0·72; *P*<0·001).

**Fig. 4:**
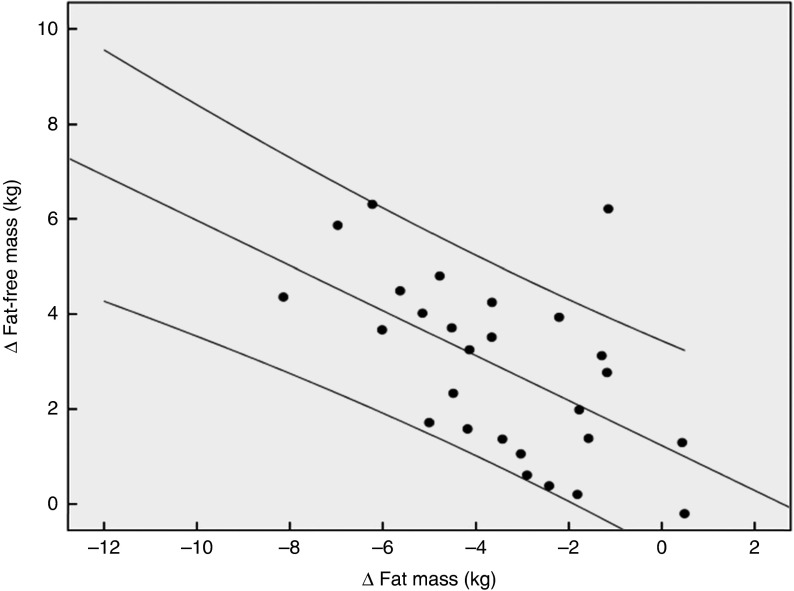
Correlation (Pearson’s *r*) between fat-free mass and fat mass changes after a 12 weeks of resistance training in elderly men (age>65 years, *n* 26) in the placebo group (*r* 0·55; *P*<0·003).

Fig. 2 demonstrates that the observed increase in FFM of 2·90 (sem 1·84) kg in the PG was more pronounced after supplementation with 15 g collagen peptides (+4·22 (sem 2·31) kg). The observed group difference was statistically significant (*P*<0·05). In addition, the decrease in FM in the collagen peptide-supplemented group (–5·45 (sem 3·17) kg) was more pronounced (*P*<0·05) compared with the PG (–3·51 (sem 2·16) kg). Although the difference was not significant, baseline characteristics showed that subjects in the PG weighed less and had relatively more FFM and less FM compared with subjects in the collagen-supplemented group. In both the groups, the loss of FM correlated with an increase in FFM; in the collagen-supplemented group, the correlation coefficient (*r* 0·72; *P*<0·001) was more pronounced than in the control group (*r* 0·55; *P*<0·003) (Figs 3 and 4).

**Fig. 5:**
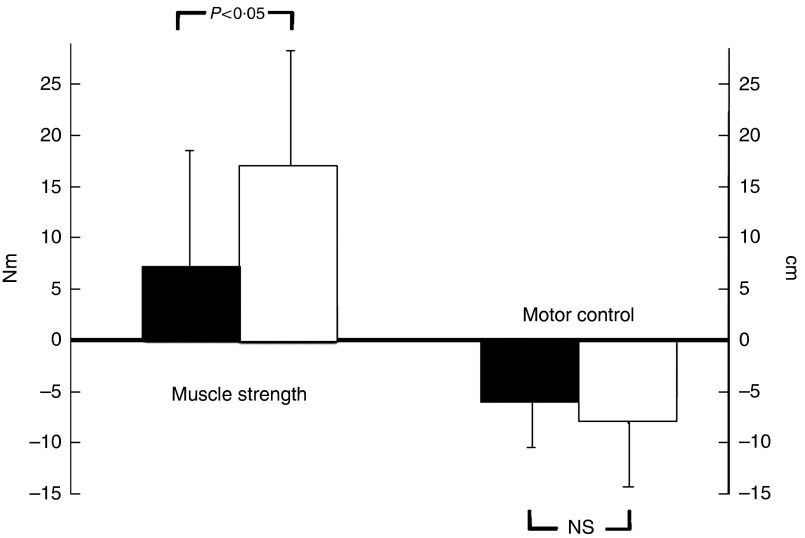
Changes in strength output and motor control after 12 weeks of resistance training referred to baseline in elderly men (age>65 years) with collagen peptide supplementation (treatment group (TG), *n* 26) or placebo (placebo group (PG), *n* 27). Values are means with their standard error of means. Significance tested by ANOVA considering time×treatment interactions. 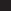, PG; 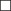, TG.

Muscle strength was increased in both the study groups after 12 weeks, but again the effect in the collagen peptide group (16·12 (sem 12·9) Nm) was more distinct than in the PG (+7·38 (sem 13·2) Nm), demonstrating a statistically significant difference (*P*<0·05) (Fig. 5). SMC was not significantly different from that of the PG (Fig. 5).

BM was significantly (*P*<0·001) increased during the course of the intervention in both the groups. The difference between the groups after the intervention did not reach significance.

The analysis of the nutritional protocols revealed that the subjects consumed a typical western diet and that they were not protein deficient (protein 16·4 (sem 4·2) % (0·91 g/kg body weight), fat 33·23 (sem 7·1) % and carbohydrates 43·8 (sem 8·7) %). Total energy intake was 7757·1 kJ/d (1854 kcal/d), which is in the lower reference level for this age group; however, underreporting cannot be ruled out in these subjects aiming at improving their body compositing. There were no significant differences between the dietary intake before and after the intervention period. Dietary intake did not differ between the groups.

## Discussion

The main finding of the present study is that collagen peptides further increased the benefits of the 3-month resistance training in older subjects with sarcopenia. Compared with placebo, subjects in the collagen-supplemented group showed a higher increase in FFM and muscle strength as well as a higher reduction in FM.

The results of the current study are in accordance with previous investigations, showing that resistance exercise improves strength, FFM, co-ordination as well as postural control in the ageing population^(^ ^25^ ^)^.

However, there is a controversial discussion as to whether the anabolic effect of resistance exercise can be further enhanced by protein supplementation, particularly in the elderly^(^ ^26^ ^,^ ^27^ ^)^. In experimental settings, it has been clearly demonstrated that the ingestion of dietary protein following resistance exercise stimulates muscle protein synthesis rates in the post-exercise period^(^ ^28^ ^,^ ^29^ ^)^. Nevertheless, the findings of RCT investigating this combined effect over a longer period of time have yielded controversial results. Although a considerable amount of well-controlled studies have reported an increase in FFM and muscle strength following the combination of resistance exercise with protein supplementation, other investigators could not confirm a synergistic effect^(^ ^8^ ^,^ ^9^ ^)^. A meta-analysis by Cermak *et al.* ^(^ ^11^ ^)^ analysed the combined effect of protein and resistance exercise in both younger and older subjects by pooling twenty-two RCT. The data indicate that protein supplementation increases the gains in FFM and muscular strength in both young and elder subjects^(^ ^11^ ^)^. Moreover, a most recent review supports the efficacy of nutritional supplementation in the treatment of sarcopenia^(^ ^30^ ^)^.

In the present investigation, the increase in FFM and muscle strength and the decrease in FM seemed to be more pronounced compared with previous reports^(^ ^11^ ^)^. This might be explained by the fact that the training was very extensive. It was designed and supervised by renowned experts in motor control and resistance training^(^ ^7^ ^)^. In addition, the workload was individually adapted throughout the study, and the main goal of the training procedure was the induction of muscular hypertrophy. Moreover, the participants of the present study were not suffering from severe sarcopenia with no signs of frailty or cachexia. Nevertheless, no significant correlation between the degree of sarcopenia and the individual response to supplementation and training in any of the parameters in the subjects investigated could be observed. All the subjects were living independently with a normal dietary pattern, and the recorded nutritional protocols revealed adequate protein intake. Nevertheless, the subjects did not perform physical exercise, particularly resistance exercise, on a regular basis (<1 h/week). This could further explain why the anabolic stimulus of resistance training induced such a positive effect in this non-frail population.

Other investigations with comparable study designs were not able to observe an efficacy of protein supplementation in combination with resistance exercise on body composition and muscle strength in the elderly^(^ ^31^ ^–^ ^33^ ^)^. Beside factors such as age, health, nutrition status of the subjects and design of the training programme, the type of dietary protein intake could also play a role. In a previous study, we could demonstrate that soya protein together with a resistance training programme, comparable with the one applied in the present investigation, increased muscle mass to a higher extent than in the control group without protein supplementation^(^ ^34^ ^)^.

The effects of collagen peptides on body composition and muscular power output have not been investigated previously. Thus far, studies have mainly focused on the effects of collagen peptides on skin health and degenerative joint diseases such as osteoarthritis^(^ ^13^ ^,^ ^14^ ^,^ ^35^ ^–^ ^40^ ^)^. The impact on body composition has not been in the focus, as it is generally believed that the relatively low biological value of collagen would not favour a significant improvement on muscular net protein synthesis. The results of the present investigation do not support this assumption, and the following findings could contribute to further explain the increase in FFM and strength following collagen peptides supplementation: it has been shown that collagen peptide intake was superior to whey protein in maintaining N balance and body weight during a low-protein diet^(^ ^18^ ^)^. Although collagen has a low protein digestibility corrected amino acid score, its N content may be higher compared with whey on a per gram basis due to a high proportion of amino acids having low molecular weight or containing more than one N atom.

Furthermore, it has been speculated that the timing of protein supplementation as well as the absorption kinetic of the administered protein can have an influence on the efficacy^(^ ^41^ ^)^. Some studies revealed that a fast digestion and rapid absorption kinetic could influence the enhancement of muscle hypertrophy by proteins. It has been proposed that the anabolic window for optimal post-exercise anabolic effects begins to close after 90**–**120 min^(^ ^42^ ^,^ ^43^ ^)^. In the present study, collagen peptides were ingested within 60 min after training. Therefore, it could be possible that the short post-exercise interval and the rapid digestibility and absorption of collagen peptides following supplementation^(^ ^15^ ^,^ ^44^ ^)^ may have supported the post-exercise muscle protein anabolism. It has to be critically remarked that a most recent meta-analysis did not support the hypothesis of this anabolic window theory^(^ ^30^ ^)^. Therefore, further research in this area including collagen peptides is necessary.

Another rather speculative explanation for the observed effects could be that collagen is rich in arginine and glycine, both known to be important substrates for the synthesis of creatine in the human body. Creatine supplementation has been shown to improve both muscle mass and muscular function in some but not all studies^(^ ^45^ ^)^. In the recent years, evidence supporting the theory suggesting that creatine supplementation may also play a role in reducing sarcopenia in aged subjects is increasing^(^ ^46^ ^)^. Therefore, it would be interesting to determine the amount of creatine in the muscle cells following collagen peptide supplementation in future studies.

In addition, Timmerman & Volpi^(^ ^47^ ^)^ discussed the positive effect of an increased microvascular perfusion, and thus increased amino acid delivery, on enhanced anabolic responses after protein supplementation. Collagen peptides have shown to positively influence microcirculation^(^ ^48^ ^,^ ^49^ ^)^; therefore, this might cause an additional beneficial effect in promoting muscle growth compared with other protein sources.

Finally, several investigations have shown that collagen peptides significantly reduce pain in subjects with osteoarthritis as well as functional joint pain^(^ ^13^ ^,^ ^50^ ^)^. Therefore, it could be speculated that the subjects who were supplemented with collagen peptides were able to perform the resistance exercises with less pain, and therefore had a better training gain.

The reason for the higher increase in muscular strength may also be related to one of the above-mentioned factors or simply goes along with the higher amount of muscle mass. The existence of a specific effect on muscular recruitment cannot be assessed on the basis of the design of the study.

Nevertheless, the study has several limitations. The statistical analysis was a completers’ analysis and not an intention-to-treat analysis. We decided to choose this approach as the dropout causes were not in direct relationship with the intervention protocol and the subjects dropped out before the final examination.

Furthermore, we chose a placebo that did not deliver any extra calories. It could be speculated that the additional amount of energy provided by the collagen peptides may be responsible for the respective effects. However, to our knowledge, there are no data showing that additional calories – for example, by carbohydrates – would favour muscle hypertrophy. Therefore, we do not think that it was simply the lower amount of calories in the control group that accounts for the differences observed. Finally, the randomisation yielded two groups with baseline differences in FM and FFM; although the difference was not statistically significant, an influence of these baseline differences on the respective results cannot be ruled out completely.

In conclusion, the findings of the present study have confirmed previous results that 60 min of resistance exercise, performed three times per week, is well suited to significantly increase muscle mass, muscular strength and motor control in subjects with sarcopenia class I or II. Moreover, the study has demonstrated that the combination of resistance exercise and collagen peptide supplementation resulted in a more pronounced improvement of body composition, as indicated by a significant increase in muscle mass and decrease in FM, compared with placebo. In addition, muscular strength was significantly improved after collagen peptide intake compared with the training programme plus placebo.

Further studies should investigate the effect of combined resistance training and collagen peptide intake in other study populations, including sex and different age groups and should focus on the mode of action as well as on the required dosage.
