# Collagen peptide supplementation in combination with resistance training improves body composition and increases muscle strength in elderly sarcopenic men: a randomised controlled trial – CORRIGENDUM

**DOI:** 10.1017/S000711452510425X

**Published:** 2025-09-14

**Authors:** Denise Zdzieblik, Steffen Oesser, Manfred W. Baumstark, Albert Gollhofer, Daniel König

**Explanation of correction:** The above article was missing the following information from the acknowledgements and conflict of interest statement.

Original text and correction:

Acknowledgements: (page no. 1244)

The authors thank the whole team of the Mooswaldklinik in Freiburg for the realisation of the training programme and the energy they have put into this project.

Part of the costs were paid by Gelita AG, Uferstraße 7, Eberbach, Germany.

D. K. was the principal investigator of the study. S. O. and M. W. B. were involved in the design and execution of the study and performed the statistical analysis. All the authors read and approved the final version of the manuscript. The planning, organisation, monitoring and analysis of the study were performed independently by the investigators.

There are no conflicts of interest.

CORRECTION:

Acknowledgements:

The authors thank the whole team of the Mooswaldklinik in Freiburg for the realisation of the training programme and the energy they have put into this project.

The research did not receive any specific grant from funding agencies, whether commercial or non-profit. Gelita AG provided the study samples and paid an amount covering the costs of participant compensation and sample analysis.

D. K. was the principal investigator of the study. S. O. and M. W. B. were involved in the design and execution of the study and performed the statistical analysis. All the authors read and approved the final version of the manuscript. The planning, organisation, monitoring and analysis of the study were performed independently by the investigators.

Conflict of interest: The author Dr. Steffen Oesser is named as a co-inventor of a patent application which has been filed on May 13, 2013 relating to the active substance mentioned in the publication. Dr. Oesser receives no royalty-payments or other remunerations in connection with the exploitation of the patent application.
